# Diagnostic Performance of Immature Platelet Fraction in Patients with Suspected Deep Vein Thrombosis

**DOI:** 10.3390/healthcare14132018

**Published:** 2026-07-07

**Authors:** Quiny Kurnia, Dimas Bayu, Haerani Rasyid, Andi Alfian Zainuddin, Syakib Bakri, Andi Makbul Aman

**Affiliations:** 1 Department of Internal Medicine, Faculty of Medicine, Hasanuddin University, Makassar 90245, Indonesia; 2 Department of Public Health and Community Medicine, Faculty of Medicine, Hasanuddin University, Makassar 90245, Indonesia

**Keywords:** deep vein thrombosis, immature platelet fraction, D-dimer, wells score, Doppler ultrasound

## Abstract

**Background/Objectives**: Deep Vein Thrombosis (DVT) remains a major cause of morbidity and mortality. Current diagnostic approaches rely on the Wells score and D-dimer, but both have limitations. Immature Platelet Fraction (IPF) has emerged as a potential biomarker reflecting platelet activation and thrombosis activity. The aim of this study was to compare the diagnostic performance of IPF, the Wells score, and D-dimer for the diagnosis of DVT confirmed by Doppler ultrasonography. **Methods**: This retrospective diagnostic accuracy study included 53 patients with suspected DVT treated at Dr. Wahidin Sudirohusodo Hospital, Makassar, Indonesia. Patients underwent clinical assessment using the Wells score, laboratory examination of D-dimer and IPF, and Doppler ultrasonography as the reference standard. Associations were analyzed using Chi-square and multivariate logistic regression. Diagnostic performance was evaluated using ROC curve analysis. **Results**: DVT was confirmed in 32 patients (60.4%). A high Wells score was associated with DVT (OR 4.762; *p* = 0.009), as was elevated D-dimer (OR 6.364; *p* = 0.005). Elevated IPF demonstrated a significant association with DVT (OR 14.000; *p* < 0.001). In multivariate analysis, IPF remained significantly associated with DVT (OR 11.819; *p* = 0.007). ROC analysis demonstrated that IPF had the highest diagnostic accuracy among individual variables (AUC 0.832), followed by D-dimer (AUC 0.753) and Wells score (AUC 0.695). Combination analysis improved diagnostic performance (AUC 0.845). **Conclusions**: IPF demonstrated promising diagnostic performance in patients with suspected DVT. Nevertheless, these findings should be considered preliminary and require confirmation in larger prospective studies before clinical implementation can be considered.

## 1. Introduction

Thrombosis is the process of blood clot formation within the vascular system that may occur in both arterial and venous circulation and remains a major cause of cardiovascular disease worldwide. From a pathophysiological perspective, arterial thrombosis is primarily triggered by rupture of atherosclerotic plaques leading to platelet activation and the formation of platelet-rich thrombi, whereas venous thrombosis is more closely associated with venous stasis, hypercoagulability, and endothelial dysfunction, resulting in fibrin- and erythrocyte-rich thrombus formation [1,2]. Deep Vein Thrombosis is one of the major manifestations of venous thromboembolism and is associated with substantial morbidity and mortality, particularly among hospitalized patients [3]. In the process of venous thrombus formation, platelets play a crucial role through prothrombotic coagulation mechanisms following vascular injury. Several studies have demonstrated that immature platelets are larger and exhibit greater thrombotic activity compared with mature platelets, thereby potentially contributing to the progression of thrombosis in patients with DVT [4].

The diagnosis of DVT is established through a stepwise approach that combines clinical assessment, laboratory testing, and imaging studies, with Doppler ultrasonography serving as the reference standard because of its high sensitivity and specificity [5]. The Wells score is commonly used to assess the initial clinical probability of DVT, whereas D-dimer testing functions as a highly sensitive screening tool to exclude DVT in patients with low to intermediate clinical probability [6]. Although this diagnostic algorithm has been widely adopted in clinical practice, the limited specificity of D-dimer highlights the need for additional biomarkers that more accurately reflect thrombotic activity. Platelets play essential roles not only in hemostasis but also in inflammation and immunothrombosis, both of which contribute to thrombus formation [3,7]. Platelet activity can be evaluated using several hematological parameters, including IPF, which reflects the proportion of circulating immature or reticulated platelets. Immature platelets are larger, metabolically more active, and exhibit greater prothrombotic potential compared with mature platelets, and elevated IPF levels have been associated with increased thrombotic risk in various cardiovascular and thromboembolic disorders [7]. Therefore, IPF has emerged as a promising additional biomarker that may improve the diagnostic evaluation of DVT beyond conventional D-dimer testing [8].

Based on this background, the present study aimed to compare the diagnostic performance of IPF with conventional diagnostic tools, including D-dimer and Wells score, in diagnosing DVT. Furthermore, this study sought to evaluate the potential utility of IPF as an additional diagnostic biomarker that may improve the accuracy of DVT detection and support more individualized therapeutic decision-making in patients with DVT.

## 2. Materials and Methods

### 2.1. Study Design and Subjects

This retrospective diagnostic accuracy study was conducted to evaluate the diagnostic performance of IPF, D-dimer, and Wells score in diagnosing DVT. The study was carried out in the Internal Medicine inpatient wards of Dr. Wahidin Sudirohusodo Hospital, Makassar, Indonesia, between February and December 2025.

A total of 53 hospitalized patients with clinical suspicion of DVT were included in this study. Subjects were recruited consecutively until the required sample size was achieved. Eligible participants were patients aged ≥18 years presenting with clinical manifestations suggestive of DVT, including unilateral lower extremity swelling, pitting edema, localized tenderness, or other signs of venous thrombosis, who had not received prior antithrombotic therapy. All subjects underwent D-dimer testing, Immature Platelet Fraction (IPF) measurement, and Doppler ultrasonography examination. Patients with thrombocytopenia or pregnancy were excluded from the study.

Clinical probability of DVT was assessed using the Wells score, which is generally categorized into low-, moderate-, and high-risk probability groups. However, in the present study, subjects were classified only into moderate- and high-risk categories. Patients with low-risk Wells score were not included because they did not undergo Doppler ultrasonography examination according to routine clinical practice, as low-risk patients with negative screening results were considered unlikely to have DVT and therefore did not require further imaging evaluation.

### 2.2. Data Collection

Demographic, clinical, and laboratory data were collected retrospectively from inpatient medical records and hospital databases. Baseline characteristics included age, sex, and comorbidities such as diabetes mellitus, cardiovascular disease, malignancy, postoperative status, and prolonged immobilization. Clinical probability of DVT was assessed using the Wells score and categorized according to established diagnostic criteria. Patients with thrombocytopenia were excluded from the study because reduced platelet counts may influence IPF measurements and potentially confound the interpretation of platelet production and turnover. None of the patients received antithrombin replacement therapy before enrollment or prior to baseline laboratory assessment. Following confirmation of DVT by Doppler ultrasonography, patients were treated according to institutional protocols, including antithrombin replacement therapy when clinically indicated.

Peripheral venous blood samples were collected at the time of hospital admission and prior to the initiation of anticoagulant therapy. Laboratory examination included D-dimer and IPF measurements. D-dimer analysis was performed using a Sysmex CA-660 coagulation analyzer (Sysmex Corporation, Kobe, Japan) based on a latex-enhanced immunoturbidimetric assay, and results were reported in fibrinogen equivalent units (FEU). IPF measurement was performed using a Sysmex XN-1000 automated hematology analyzer (Sysmex Corporation, Kobe, Japan), which utilizes fluorescence flow cytometry technology to identify immature platelets according to their higher ribonucleic acid (RNA) content compared with mature platelets. Following fluorescent staining of platelet RNA, platelets were analyzed based on fluorescence intensity and light scatter characteristics, and IPF was reported as the percentage of immature platelets among the total platelet population.

D-dimer levels > 0.5 μg/mL FEU were categorized as elevated, whereas IPF values > 6.0% were considered increased. The IPF cut-off value was selected based on the upper limit of the internationally accepted reference range for healthy adults (1.1–6.1%) and institutional laboratory standards. Doppler ultrasonography was performed in all subjects by experienced radiologists and served as the reference standard for DVT diagnosis. A whole-leg ultrasound approach was used to evaluate the deep venous system of the lower extremities. The radiologists interpreting the examinations were blinded to the IPF, D-dimer, and Wells score results. DVT was considered present when thrombus was identified on ultrasonographic examination. Based on the ultrasonographic findings, subjects were subsequently classified into DVT-positive and DVT-negative groups.

### 2.3. Statistical Analysis

Statistical analyses were performed using SPSS software version 26.0 (IBM Corp., Armonk, NY, USA). Descriptive statistics were used to summarize baseline characteristics and are presented as mean ± standard deviation (SD) for continuous variables and frequency (percentage) for categorical variables. Comparisons between groups were analyzed using the Chi-square test or Fisher’s exact test, as appropriate. Odds ratios (ORs) and 95% confidence intervals (CIs) were calculated to assess the strength of association between clinical variables and DVT occurrence.

Multivariable logistic regression analysis was performed to evaluate the association of IPF with DVT after adjustment for established diagnostic variables. Based on their clinical relevance and their role in DVT assessment, IPF, D-dimer, and Wells score were included in the multivariable model. Given the exploratory nature of the study and the limited sample size, the results of the multivariable analysis should be interpreted with caution.

Receiver Operating Characteristic (ROC) curve analysis was conducted to evaluate the diagnostic performance of the Wells score, D-dimer, IPF, and their combined model. Diagnostic accuracy was assessed using the area under the curve (AUC), sensitivity, specificity, and optimal cut-off values. A two-tailed *p*-value < 0.05 was considered statistically significant throughout the study.

## 3. Results

### 3.1. Baseline Characteristics of the Study

A total of 53 patients with suspected DVT were included in this study. Female patients were more predominant than males (56.6% vs. 43.4%), and most subjects were aged >50 years (54.7%). Doppler ultrasonography confirmed thrombus formation in 32 patients (60.4%). Based on the Wells score assessment, the majority of patients were classified as high-risk (64.2%). Elevated IPF and D-dimer levels were observed in 66.0% and 73.6% of patients, respectively. Type 2 diabetes mellitus was the most common comorbidity (54.7%), followed by malignancy (26.4%) and cardiovascular disease (20.8%). Baseline characteristics of the study population are summarized in Table 1.

### 3.2. Association Between Wells Score and the Occurrence of DVT

Table 2 shows the association between the Wells score and the occurrence of DVT. A high-risk Wells score was significantly associated with DVT occurrence, with patients demonstrating approximately 4.7-fold higher odds of DVT compared with those in the moderate-risk group (OR 4.762; 95% CI 1.429–15.872; *p* = 0.009).

### 3.3. Association Between D-Dimer Levels and the Occurrence of DVT

Table 3 shows the association between D-dimer levels and the occurrence of DVT. Elevated D-dimer levels were significantly associated with DVT occurrence, with patients demonstrating approximately 6.3-fold higher odds of DVT compared with those with normal D-dimer levels (OR 6.364; 95% CI 1.645–24.624; *p* = 0.005).

### 3.4. Association Between IPF and the Occurrence of DVT

Table 4 shows the association between IPF and the occurrence of DVT. Elevated IPF levels were significantly associated with DVT occurrence, with patients demonstrating approximately 14-fold higher odds of DVT compared with those with normal IPF levels (OR 14.000; 95% CI 3.501–55.978; *p* < 0.001).

### 3.5. Multivariate Analysis of Factors Associated with DVT

Table 5 shows the results of the multivariate logistic regression analysis for factors associated with DVT occurrence. Elevated IPF remained significantly associated with DVT after adjustment for other variables (OR 11.819; 95% CI 1.969–70.957; *p* = 0.007). In contrast, elevated D-dimer levels (OR 2.998; 95% CI 0.532–16.907; *p* = 0.213) and high-risk Wells score (OR 0.717; 95% CI 0.106–4.867; *p* = 0.734) were not significantly associated with DVT in the multivariate model.

### 3.6. ROC Curve Analysis and Diagnostic Performance of Wells Score, D-Dimer, IPF, and Their Combination for Diagnosing DVT

**Figure 1:**
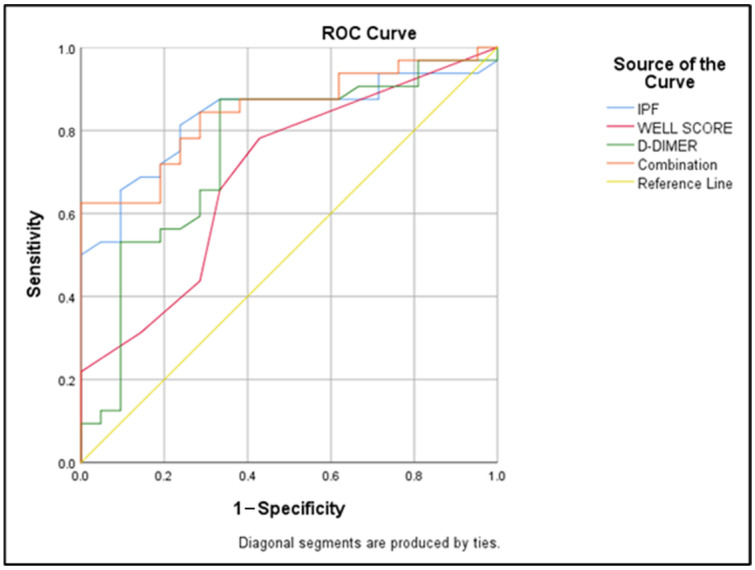
Receiver Operating Characteristic curves for Wells score (AUC = 0.695, 95% CI 0.550–0.840), D-dimer (AUC = 0.753, 95% CI 0.613–0.893), IPF (AUC = 0.832, 95% CI 0.720–0.944), and the combined (AUC = 0.845, 95% CI 0.742–0.949) model in the diagnosis of deep vein thrombosis. The combined model was derived from the multivariable logistic regression model incorporating Wells score, D-dimer, and IPF.

As summarized in Table 6 and illustrated in Figure 1 the ROC curve analysis evaluating the diagnostic performance of Wells score, D-dimer, IPF, and their combination for diagnosing DVT. Among the individual variables, IPF demonstrated the highest diagnostic accuracy with an AUC of 0.832, followed by D-dimer (AUC = 0.753) and Wells score (AUC = 0.695). Furthermore, the combined model of Wells score, D-dimer, and IPF showed improved diagnostic performance with an AUC of 0.845, sensitivity of 78.1%, and specificity of 76.2%.

## 4. Discussion

Deep Vein Thrombosis remains a major clinical challenge because of its high morbidity and mortality, particularly among hospitalized patients. Early diagnosis is essential to prevent severe complications such as pulmonary embolism and post-thrombotic syndrome. Current diagnostic strategies commonly rely on clinical prediction models, including the Wells score, combined with laboratory biomarkers such as D-dimer and confirmatory imaging using Doppler ultrasonography [9]. Previous studies have demonstrated that the integration of the Wells score and D-dimer improves diagnostic accuracy and reduces unnecessary imaging examinations [10]. However, the limited specificity of D-dimer and the subjective nature of clinical scoring systems continue to encourage the exploration of additional biomarkers that more accurately reflect thrombotic activity.

In the present study, a significant association was observed between the Wells score and DVT occurrence. Patients classified as high-risk according to the Wells score demonstrated approximately 4.7-fold higher odds of DVT compared with those in the moderate-risk group. These findings are consistent with the meta-analysis by Goodacre et al., which reported that patients with a high Wells score had a significantly increased likelihood of DVT, supporting the clinical utility of the Wells score as an initial predictive tool in DVT diagnosis [11]. Similarly, Modi et al. demonstrated good discriminative ability of the Wells score with an AUC of 0.78, indicating acceptable diagnostic performance in distinguishing patients with and without DVT [12]. Furthermore, Silveira et al. reported that the Wells score remained clinically relevant even among hospitalized patients, with an AUC of approximately 0.72 [13]. These findings support the continued role of the Wells score as an accessible and practical tool for initial risk stratification in patients with suspected DVT.

This study also demonstrated a significant relationship between elevated D-dimer levels and DVT occurrence, with patients showing approximately 6.3-fold increased odds of DVT. These results are consistent with previous studies demonstrating the diagnostic importance of D-dimer in venous thromboembolism. A meta-analysis by Heim et al. reported that D-dimer testing possesses high diagnostic odds ratios and excellent sensitivity for detecting DVT, making it useful as an initial screening test [14]. Likewise, Fancher et al. showed that although D-dimer has a sensitivity exceeding 90%, its specificity remains limited because elevated levels may occur in various inflammatory and systemic conditions [15]. This limitation was also reflected in the present study, where elevated D-dimer levels were observed not only in DVT-positive patients but also among a substantial proportion of non-DVT subjects. Moreover, Imai et al. demonstrated that elevated D-dimer independently predicted DVT occurrence in multivariate analysis [16]. Nevertheless, in the current study, D-dimer lost statistical significance after multivariable adjustment, suggesting that additional biomarkers may contribute complementary diagnostic information in the assessment of DVT.

The most important finding of this study was the strong association between IPF and DVT occurrence. Elevated IPF levels were associated with a 14-fold increased risk of DVT and remained significantly associated with DVT after multivariate adjustment. These findings support the growing evidence that platelet activation and turnover play central roles in venous thrombosis. Immature platelets are larger, metabolically more active, and possess greater prothrombotic potential than mature platelets, thereby contributing to thrombus propagation and vascular inflammation [17]. Elevated IPF reflects increased thrombopoietic activity and the release of reticulated platelets into circulation, which may indicate ongoing thrombotic processes in DVT patients.

Our findings are consistent with previous studies investigating platelet-related biomarkers in thromboembolic diseases. Schorling et al. demonstrated that elevated platelet parameters, including IPF, were associated with venous thromboembolism among cancer patients [8]. Similarly, a systematic review by Malte et al. reported that increased IPF was significantly associated with higher thrombotic risk, further supporting its role as a thrombotic biomarker [18]. Brin et al. also demonstrated that elevated IPF was associated with poorer thrombotic outcomes in pulmonary embolism patients and retained predictive significance in regression analysis [19]. Collectively, these studies reinforce the biological plausibility of IPF as a marker of thrombotic activity and support its potential role in DVT diagnosis.

Receiver Operating Characteristic analysis further demonstrated that IPF had the highest diagnostic accuracy among the evaluated variables, with an AUC of 0.832, compared with D-dimer and the Wells score. Moreover, the combination of Wells score, D-dimer, and IPF yielded the best overall diagnostic performance, with an AUC of 0.845, a sensitivity of 78.1%, and a specificity of 76.2%. These findings indicate that combining clinical assessment with laboratory biomarkers improves diagnostic discrimination compared with individual parameters alone. Previous studies by Sartori et al. demonstrated that combining the Wells score and D-dimer improved diagnostic accuracy for DVT [20]. Likewise, Lesmana et al. reported that integration of the Wells score with D-dimer significantly enhanced diagnostic sensitivity and overall predictive performance [21]. The present study expands upon these findings by demonstrating that the addition of IPF further improves diagnostic capability.

From a clinical perspective, these findings have important implications. While Doppler ultrasonography remains the diagnostic reference standard, imaging may not always be readily available, particularly in resource-limited settings. Therefore, the identification of accessible hematological biomarkers such as IPF may help optimize early risk stratification and guide decision-making regarding further imaging evaluation. Compared with D-dimer, which is highly sensitive but less specific, IPF may better reflect active thrombotic processes and platelet-driven immunothrombosis. Consequently, combining the Wells score, D-dimer, and IPF may provide a more comprehensive and clinically practical diagnostic approach for patients with suspected DVT.

Several limitations should be acknowledged. First, the retrospective single-center design may limit the generalizability of the findings. Second, the relatively small sample size, without a priori sample size calculation, resulted in wide confidence intervals and reduced statistical precision; therefore, the study should be considered exploratory and hypothesis-generating. Third, the exclusion of patients with low-risk Wells scores, due to the lack of routine Doppler ultrasonography in this group, may have introduced spectrum bias and contributed to the high DVT prevalence observed, limiting the applicability of the findings to unselected populations with suspected DVT. Fourth, NSAID use was not systematically assessed and was not included as an exclusion criterion, potentially influencing IPF measurements. Fifth, several relevant clinical and laboratory confounders, including inflammatory conditions, renal dysfunction, hematological disorders, active bleeding, and medications affecting platelet function, were not systematically evaluated, and residual confounding cannot be excluded. Furthermore, internal validation procedures such as bootstrapping or cross-validation were not performed, and the stability of the multivariable model requires confirmation in larger prospective cohorts. In addition, although ROC curve analysis was performed to evaluate the diagnostic performance of IPF, D-dimer, Wells score, and their combined model, no formal statistical comparison between the AUCs (e.g., DeLong’s test) was conducted. Therefore, while IPF demonstrated the highest observed AUC, it cannot be concluded that its diagnostic performance was statistically superior to the other evaluated parameters. This limitation should be considered when interpreting the comparative diagnostic performance of the investigated biomarkers. Despite these limitations, this study provides preliminary evidence supporting the diagnostic value of IPF in DVT and highlights its potential as a promising adjunctive biomarker for further investigation.

## 5. Conclusions

In conclusion, this retrospective single-center diagnostic accuracy study of hospitalized patients with moderate- or high-risk Wells score demonstrated that IPF showed the highest AUC among the evaluated diagnostic variables for the identification of DVT. These findings suggest that IPF may serve as a promising adjunctive biomarker in the assessment of selected patients with suspected DVT. However, the study population was highly selected, the sample size was relatively small, and no formal statistical comparison between ROC curves was performed. In addition, the findings have not undergone external validation and should therefore be considered exploratory and hypothesis-generating. Larger, prospective, multicenter studies are required to confirm the diagnostic utility of IPF and to determine its potential role in routine DVT assessment.

## Figures and Tables

**Table 1:** Baseline Characteristics of the Study.

Variables	Categories	n	%
Sex	Male	23	43.4
	Female	30	56.6
Doppler Ultrasonography Findings	Thrombus (−)	21	39.6
	Thrombus (+)	32	60.4
Age	≤50 years	24	45.3
	>50 years	29	54.7
Wells Score	Moderate risk	19	35.8
	High risk	34	64.2
IPF	Normal	18	34
	Elevated	35	66
D-dimer	Normal	14	26.4
	Elevated	39	73.6
Comorbidities	Type 2 Diabetes Mellitus	29	54.7
	Cardiovascular disease	11	20.8
	Malignancy	14	26.4
	Other comorbidities *	7	13.2

* Other comorbidities included postoperative status (n = 4) and prolonged immobilization (n = 3). IPF: Immature Platelet Fraction.

**Table 2:** Association Between Wells Score and the Occurrence of DVT.

Doppler Ultrasonography Findings	High-Risk Wells Score n (%)	Moderate-Risk Wells Score n (%)	Total n (%)	*p*-Value	OR	95% CI
Thrombus (+)	25 (78.1)	7 (21.9)	32 (100.0)	0.009	4.762	1.429–15.872
Thrombus (−)	9 (42.9)	12 (57.1)	21 (100.0)			

Chi-square test, *p* < 0.05.

**Table 3:** Association Between D-Dimer Levels and the Occurrence of DVT.

Doppler Ultrasonography Findings	Elevated D-Dimer n (%)	Normal D-Dimer n (%)	Total n (%)	*p*-Value	OR	95% CI
Thrombus (+)	28 (87.5)	4 (12.5)	32 (100.0)	0.005	6.364	1.645–24.624
Thrombus (−)	11 (52.4)	10 (47.6)	21 (100.0)			

Chi-square test, *p* < 0.05.

**Table 4:** Association Between IPF and the Occurrence of DVT.

Doppler Ultrasonography Findings	Elevated IPF n (%)	Normal IPF n (%)	Total n (%)	*p*-Value	OR	95% CI
Thrombus (+)	28 (87.5)	4 (12.5)	32 (100.0)	<0.001	14.000	3.501–55.978
Thrombus (−)	7 (33.3)	14 (66.7)	21 (100.0)			

Chi-square test, *p* < 0.05. IPF: Immature Platelet Fraction.

**Table 5:** Multivariate Logistic Regression Analysis of Factors Associated with DVT.

Variables	*p*-Value	OR	95% CI
Elevated IPF	0.007	11.819	1.969–70.957
Elevated D-dimer	0.213	2.998	0.532–16.907
High-risk Wells score	0.734	0.717	0.106–4.867

IPF: Immature Platelet Fraction; OR: Odds Ratio; CI: Confidence Interval.

**Table 6:** Diagnostic Performance of Wells Score, D-Dimer, IPF, and Their Combination Based on ROC Analysis.

Variables	Cut-Off Value	Thrombus (+), n (%)	*p*-Value	AUC	Specificity (%)	Sensitivity (%)	95% CI
Wells Score	3	25 (78.1)	0.009	0.695	66.7	65.6	0.550–0.840
D-Dimer	2.28 μg/mL	21 (65.6)	0.021	0.753	66.7	65.6	0.613–0.893
IPF	6.35%	28 (81.3)	<0.001	0.832	76.2	75	0.720–0.944
Combination Model	–	32 (60.4)	<0.001	0.845	76.2	78.1	0.742–0.949

ROC: Receiver Operating Characteristic; AUC: Area Under the Curve; IPF: Immature Platelet Fraction; CI: Confidence Interval.

## Data Availability

The datasets associated with the present study are available upon reasonable request by interested researchers.

## Abbreviations

The following abbreviations are used in this manuscript:

IPF	Immature Platelet Fraction
DVT	Deep Vein Thrombosis
FEU	Fibrinogen Equivalent Units
ROC	Receiver Operating Characteristic
OR	Odds Ratio

## References

[1] Esmon C.T. (2009). Basic mechanisms and pathogenesis of venous thrombosis. Blood Rev..

[2] Yamashita A., Asada Y. (2023). Underlying mechanisms of thrombus formation/growth in atherothrombosis and deep vein thrombosis. Pathol. Int..

[3] Cay N., Ipek A., Gumus M., Birkan Z., Ozmen E. (2012). Platelet activity indices in patients with deep vein thrombosis. Clin. Appl. Thromb..

[4] Heestermans M., Poenou G., Duchez A.C., Hamzeh-Cognasse H., Bertoletti L., Cognasse F. (2022). Immunothrombosis and the Role of Platelets in Venous Thromboembolic Diseases. Int. J. Mol. Sci..

[5] Zierler B.K. (2004). Ultrasonography and Diagnosis of Venous Thromboembolism. Circulation.

[6] Price C.P., Fay M., Hopstaken R.M. (2021). Point-of-Care Testing for D-Dimer in the Diagnosis of Venous Thromboembolism in Primary Care: A Narrative Review. Cardiol. Ther..

[7] Handtke S., Thiele T. (2020). Large and small platelets—(When) do they differ?. J. Thromb. Haemost..

[8] Schorling R.M., Pfrepper C., Golombek T., Cella C.A., Muñoz-Unceta N., Siegemund R., Engel C., Petros S., Lordick F., Knödler M. (2020). Evaluation of Biomarkers for the Prediction of Venous Thromboembolism in Ambulatory Cancer Patients. Oncol. Res. Treat..

[9] Khan M.M., Begum M., Anees A., Mishra A. (2024). Advancements in Understanding and Managing Deep Vein Thrombosis: A Contemporary Perspective. Int. J. Pharm. Drug Anal..

[10] Zaid N.A., Desoky M.S., El Attia S.F. (2020). Reducing ultrasound in diagnosing deep vein thrombosis by using clinical scores and D-dimer testing. Int. Surg. J..

[11] Goodacre S., Sutton A.J., Sampson F.C. (2005). Meta-analysis: The value of clinical assessment in the diagnosis of deep venous thrombosis. Ann. Intern. Med..

[12] Modi S., Deisler R., Gozel K., Reicks P., Irwin E., Brunsvold M., Banton K., Beilman G.J. (2016). Wells criteria for DVT is a reliable clinical tool to assess the risk of deep venous thrombosis in trauma patients. World J. Emerg. Surg..

[13] Silveira P.C., Ip I.K., Goldhaber S.Z., Piazza G., Benson C.B., Khorasani R. (2015). Performance of Wells score for deep vein thrombosis in the inpatient setting. JAMA Intern. Med..

[14] Heim S.W., Schectman J.M., Siadaty M.S., Philbrick J.T. (2004). D-dimer testing for deep venous thrombosis: A metaanalysis. Clin. Chem..

[15] Fancher T.L., White R.H., Kravitz R.L. (2004). Combined use of rapid D-dimer testing and estimation of clinical probability in the diagnosis of deep vein thrombosis: Systematic review. BMJ.

[16] Imai N., Miyasaka D., Shimada H., Suda K., Ito T., Endo N. (2017). Usefulness of a novel method for the screening of deep vein thrombosis by using a combined D-dimer-and age-based index before total hip arthroplasty. PLoS ONE.

[17] Gumiężna K., Baruś P., Sygitowicz G., Wiśniewska A., Ochijewicz D., Pasierb K. (2023). Immature platelet fraction in cardiovascular diagnostics and antiplatelet therapy monitoring. Cardiol. J..

[18] Malte A.L., Højbjerg J.A., Larsen J.B. (2024). Platelet parameters as biomarkers for thrombosis risk in cancer: A systematic review and meta-analysis. Seminars in Thrombosis and Hemostasis.

[19] Brin A., Loutati R., Tvito A., Taha L., Karmi M., Deeb D., Manassra M., Fink N., Sabouret P., Rana J.S. (2026). Immature platelet fraction as a prognostic marker in patients with pulmonary embolism. Platelets.

[20] Sartori M., Cosmi B., Legnani C., Favaretto E., Valdré L., Guazzaloca G., Rodorigo G., Cini M., Palareti G. (2012). The Wells rule and D-dimer for the diagnosis of isolated distal deep vein thrombosis. J. Thromb. Haemost..

[21] Lesmana A., Pratama D., Wangge G. (2017). Comparison of Wells Score, D–Dimer and Combination of Wells Score and D–Dimer with Venous Duplex Ultrasonography in Diagnosis of Acute Deep Vein Thrombosis in Lower Extremity. New Ropanasuri J. Surg..

